# Differentiation of Pelvic Osteosarcoma and Ewing Sarcoma Using Radiomic Analysis Based on T2-Weighted Images and Contrast-Enhanced T1-Weighted Images

**DOI:** 10.1155/2020/9078603

**Published:** 2020-05-11

**Authors:** Yi Dai, Ping Yin, Ning Mao, Chao Sun, Jiangfen Wu, Guanxun Cheng, Nan Hong

**Affiliations:** ^1^Department of Radiology, Peking University People's Hospital, Beijing, China 100044; ^2^Department of Radiology, Peking University Shenzhen Hospital, Shenzhen, Guangdong, China 518036; ^3^Department of Radiology, Yantai Yuhuangding Hospital, Yantai, Shandong, China 264000.; ^4^GE Life Science China, Beijing, China 100000

## Abstract

**Objective:**

To determine if osteosarcoma (OS) and Ewing sarcoma (EWS) of the pelvis based on MRI can be differentiated using radiomic analysis.

**Materials and Methods:**

In this study, 3.0 T magnetic resonance (MR) data of 66 patients (40 males and 26 females, mean age 27.6 ± 13.9 years) with pathologically confirmed OS or EWS of the pelvis (35 with OS and 31 with EWS) taken from April 2013 to December 2017 were retrospectively reviewed. T2-weighted fat-saturated (T2-FS) and contrast-enhanced T1-weighted (CET1) images were manually segmented, and imaging features were extracted. Independent-sample *t*-test, Spearman's test, and the least absolute shrinkage and selection operator (LASSO) method were used to select the most useful features from the original data set. The performance of radiomic analysis was investigated by the area under the receiver operating characteristic (ROC) curve (AUC) analysis.

**Results:**

385 initial features were extracted from T2-FS and CET1 MR data. Nine features from T2-FS and 7 features from CET1 were selected by using the LASSO method. The radiomic analysis to differentiate OS and EWS of the pelvis based on T2-FS and CET1 images using the aforementioned selected features achieved AUC values of 0.881 (95% confidence interval (CI): 0.799–0.963) and 0.765 (95% CI: 0.652–0.878), respectively.

**Conclusion:**

Radiomic analysis showed potential in differentiating OS from EWS of the pelvis, in which T2-FS demonstrated better diagnostic value. To differentiate OS from EWS of the pelvis using our multiparametric MRI-based radiomic analysis could preoperatively improve diagnostic accuracy and greatly contribute to therapy planning.

## 1. Introduction

Osteosarcoma (OS) is the most common primary malignant bone tumor in children and young adults, although it has a special bimodal age distribution in the second decade of life and late adulthood [1]. OS has a predilection for the metaphyseal portions of long bones, such as the femur, tibia, and humerus [2]. Ewing sarcoma (EWS) is a high-grade sarcoma arising both in skeletal and extraskeletal locations; it is the third most common primary bone sarcoma, following OS and chondrosarcoma [3]. Moreover, EWS occurs predominantly in the bones of extremities and pelvis of children and young adolescents. OS and EWS of the pelvis share many characteristics and features compared with those arising in other parts of the body. Given the deeper location in the body than those in the extremities, pelvic malignancies are usually larger when being diagnosed, thereby greatly affecting treatment and outcome in consequence. Currently, most institutes treat OS and EWS patients with neoadjuvant chemotherapy and subsequent wide surgical resection of the tumor. However, preoperation treatment is different, in which additional adjuvant chemotherapy is usually applied to high-grade OS, whereas radiation therapy is used in EWS [1]. Owing to different treatment strategies, the need for early differential diagnosis exists. To conduct a definite diagnosis, biopsy is necessary, although it cannot overcome its invasiveness, sampling error, and potentially tumor spread. A noninvasive reliable imaging technique which helps distinguish OS from EWS is needed as a supplementary differentiated method.

Owing to the superiority of soft tissue resolution, magnetic resonance imaging (MRI) is now becoming a preferred tool for diagnosing, staging, and monitoring lesions that originate from bones and soft tissues, because it could precisely demonstrate the extent and the size of the tumor. Given that such osseous lesions are inconspicuously visible within fatty marrow on T1-weighted sequences, T2-weighted fat-saturated (T2-FS) sequences and contrast-enhanced T1-weighted (CET1) sequences have higher sensitivity in depicting lesion morphology and the surrounding involving structures. Many advanced techniques and sequences that may help in diagnosing both osseous and soft tissue tumors have been established [4, 5]; however, they may sometimes fail to provide a clear differentiation, especially for OS and EWS because of their clinical and morphological similarities.

The emergence of radiomics provides new methods on differentiation, staging, monitoring tumors, and even detecting tumor genetics. Radiomics has greatly broadened the scope of conventional medical imaging in clinical oncology, as opposed to only focusing on morphology as before. Radiomics assumes that medical images contain much vital underlying pathophysiology information by converting them into mineable high-dimensional data that could be correlated with clinical outcomes and for further use in clinical decision support [6]. In recent years, radiomics has been mainly used in oncology for staging malignant tumors [7], prediction of lymph node metastasis [8, 9], tumor prognosis [10], prediction of treatment response [11], or even prediction of the cancer phenotype [12, 13].

In the present study, for the first time, we aim to preliminarily evaluate the capability of radiomic analysis to differentiate OS from EWS of the pelvis.

## 2. Materials and Methods

### 2.1. Study Population

In this institutional review and board-approved retrospective study, informed consent was waived. The clinical and pathology database was reviewed to identify patients, and the inclusion criteria were as follows: (a) patients who underwent histological biopsy or tumor resection with tumor tissues from the pelvis that were pathologically confirmed as OS or EWS, (b) patients who underwent MRI at our institute (including T2-FS and CET1), and (c) MR data acquired using the same system from the same vendor (to minimize variations in image quality and signal intensities). The exclusion criteria were as follows: (a) poor imaging quality including obvious artifacts, (b) insidious lesions that were poorly displayed on acquired images, and (c) missing images or relevant sequences. Clinical data (age, gender, and so on) were obtained by reviewing the medical records. After adhering to these criteria, the study data set of 66 patients (40 males and 26 females, age 10–87 years, mean 27.6 ± 13.9 years) from April 2013 to December 2017 was finalized. These 66 histologically confirmed cases included 35 confirmed OS and 31 confirmed EWS.  Table 1 lists the characteristics of patients in this study. We identified the primary tumors as the locations of lesions for the large lesions that involve more than one site.

### 2.2. MR Data Acquisition

MR images were all acquired using a Discovery MR750 3.0 T scanner (GE Healthcare, Milwaukee, WI, U.S.) with an eight-channel phased array body coil for signal reception. Images were acquired by spin echo and T1-weighted 3D gradient recalled echo with a 2-point Dixon fat/water separation method (LAVA FLEX). In each study, a T2-FS sequence and a CET1 sequence were included. Other sequences such as T1-weighted fat-saturated images and general T1-weighted and T2-weighted images without fat saturation were excluded for analysis due to the retrospective data imperfection. Intravenous administration was gadodiamide injection (Omniscan®, GE Healthcare, Ireland) using a weight-based dosing protocol (0.1 mmol/L per kg body weight), and the injection rate was 2.5 mL/s. All sequence parameters are described in Table 2. All MR data were retrieved from the picture archiving and communication system (PACS) of our institute and saved in the DICOM format for further analysis.

### 2.3. Preprocessing of MR Images

Prior to radiomic analysis, all images were transferred into itk-SNAP software (version 3.6.0, http://www.itksnap.org/) for segmentation. All lesions were segmented manually by a radiologist with more than 7 years of MRI diagnostic experience. Another radiologist with more than 10 years of MRI diagnostic experience was asked for confirming the accuracy of segmentation. A revise was made by two radiologists in case of disagreement on specific image segmentation after discussion. Delineating the ROI, including the entire tumor, peritumoral edema, and periosteal reactions, was performed on one image of each sequence. The chosen slice or image demonstrated the maximum anteroposterior diameter of the lesion.

### 2.4. Radiomic Feature Extraction

Imaging features were extracted using Analysis Kit software (A.K., GE Healthcare) from T2-FS and CET1 data with manually segmented ROIs. Statistical analysis was used to perform radiomic feature extraction, which included histogram features, form factor features, Haralick features [14], gray-level cooccurrence matrix (GLCM) features (offset 1/4/7) [14], and gray-level run length matrix (GLRLM) features (offset 1/4/7, [15–17]). Some features are reported in Table 3.

### 2.5. Feature Selection Method

We used independent-sample *t*-test, Spearman's test, and the LASSO method to select the most useful features associated with the differentiation of OS and EWS from the original data set [19]. A *p* of 0.05 was considered statistically significant in independent-sample *t*-test and Spearman's test. The principle of feature selection by the LASSO method is to restrain some feature coefficients to zero by adjusting the parameter *λ*. Then, the area under the receiver operating characteristic curve (AUC) could be achieved versus log(*λ*) by using tenfold cross-validation. The LASSO method has an advantage of analyzing a large number of radiomic features with sparser samples [20]. The result derived from LASSO is usually robust and easy to be interpreted.

### 2.6. Statistical Analysis

We used R software (R Foundation for Statistical Computing, Vienna, Austria. URL: http://www.R-project.org) to perform the statistical analysis. The “glmnet” package was used for LASSO logistic regression. A *p* of 0.05 was considered statistically significant.

## 3. Results

### 3.1. Radiomic Feature Extraction/Selection

**Figure 1:**
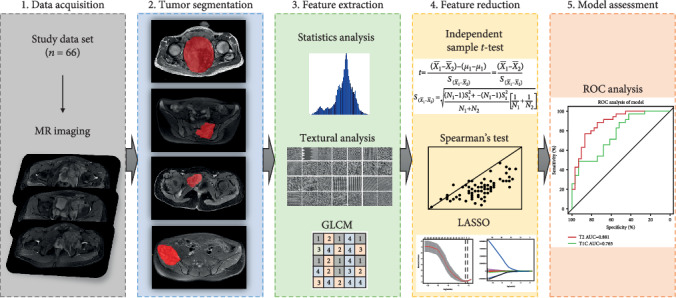
Workflow of this study. [1] MR images acquired from a qualified study data set. [2] Tumor segmentation was performed on T2-FS and CET1 MR images. Experienced radiologists contoured the tumor areas on MRI slices. [3] 385 features in total were extracted from original MR data. [4] Independent-sample *t*-test, Spearman's test, and the LASSO regression were used to conduct feature reduction. [5] ROC analysis was used to evaluate the established model.

The study flowchart is presented in Figure 1. A total of 385 initial features in total were extracted from original MR data. They were divided into five types, including histogram features (*n* = 42), form factor features (*n* = 9), Haralick features (*n* = 10), GLCM features (offset 1/4/7) (*n* = 144), and GLRLM features (offset 1/4/7) (*n* = 180). By using independent-sample *t*-test, Spearman's test, and the LASSO method, potential predictors were selected, as described in Table 4(a).

**Figure 2:**
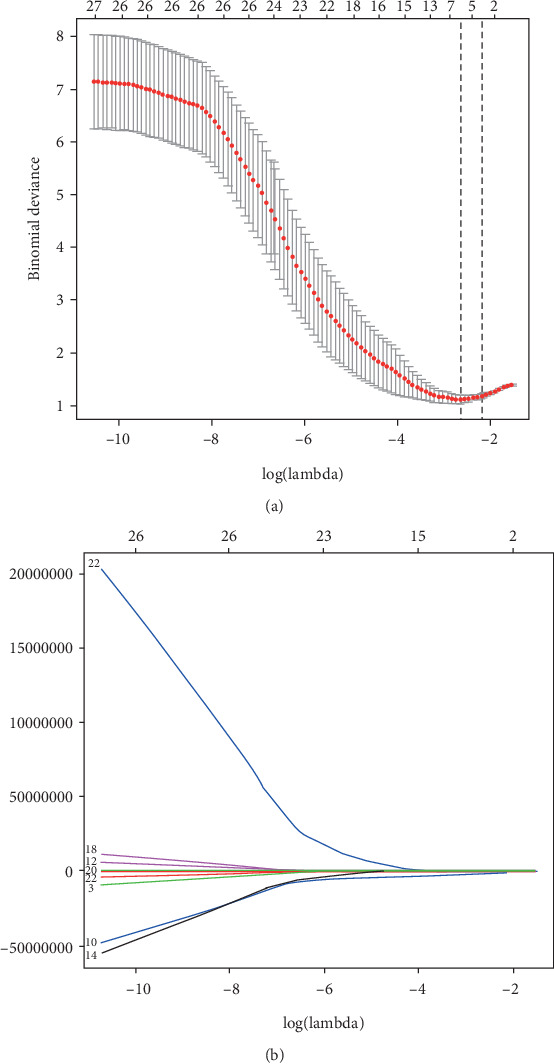
Radiomic features derived from T2-FS selected by using the least absolute shrinkage and selection operator (LASSO) binary logistic regression model. (a) Selection of the tuning parameter (*λ*) in the LASSO model via tenfold cross-validation based on minimum criteria. The lower *x*-axis indicates the log(*λ*), the upper *x*-axis indicates the number of features, and the *y*-axis indicates binomial deviances. Dotted vertical lines indicate the deviance values for each model with a given *λ*. The vertical black dotted lines define the optimal values of *λ*. A *λ* value of 0.07, with log(*λ*), -0.11 is chosen. (b) LASSO coefficient profiles of the 385 texture features. The nine selected features with nonzero coefficients are indicated in the plot.

We mainly focused on the analysis of the features extracted by using the LASSO method because the number of features extracted by the other two methods was relatively large, thereby increasing the difficulty for further analysis. Of these, by using the LASSO method, we selected nine features from T2-FS images and seven features from CET1 images that were the most powerful for the distinction between OS and EWS (Figure 2). The selection result is shown in Table 4(b).

### 3.2. Differentiated Performance

**Figure 3:**
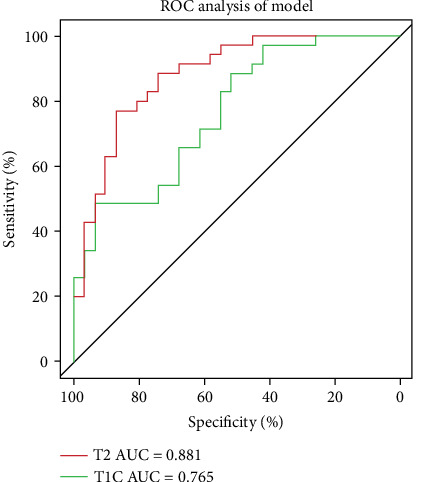
Performance of radiomic analysis using T2-FS and CET1 images. The AUC values are 0.881 (95% CI: 0.799–0.963) and 0.765 (95% CI: 0.652–0.878).

The sensitivity and specificity of the distinction between OS and EWS using data from T2-FS and CET1 are shown in Table 5. The radiomic models based on T2-FS and CET1 images achieved the AUC values of 0.881 (95% CI: 0.799-0.963) and 0.765 (95% CI: 0.652-0.878), respectively, by using DeLong's test, which are demonstrated in Figure 3.

## 4. Discussion

In this paper, we assessed the ability of our newly established radiomic model based on using multiparametric MR data to help differentiate OS from EWS of the pelvis. We evaluated 16 features that were extracted and selected by using the LASSO method. Our radiomic model yielded favorable results and constituted a new technique for the discrimination of OS and EWS. The AUC was high for both T2-FS and CET1. High specificity was achieved when using data both from T2-FS and CET1 (82.9% and 100%, respectively), and the sensitivity was also high from T2-FS (74.2%). In brief, we believe that the methodology developed in this work may serve as a reliable additional tool for differentiation OS from EWS.

Preoperative discrimination between OS and EWS of the pelvis is difficult for clinical practice, although it could be performed by using clinical data, such as age, gender, and other accessory examinations, such as medical images. OS is the most common primary malignant bone tumor, which originates from primitive bone-forming mesenchymal cells. It has a bimodal age distribution, which is during adolescence and older adulthood, respectively. OS of the pelvis is rarely seen and only accounts approximately 8% of all sites [21]. EWS of bones represents the third most common primary bone malignancy, which exceeded in prevalence only by OS and chondrosarcoma. The incidence rate peaks within the second decade of life. In terms of tumor location, EWS is commonly seen at diaphysis of long bones and the pelvis, though the most common site is still the extremities, which is quite similar as OS [22].

Both OS and EWS demonstrate aggressive features at radiography, reflecting their high-grade nature of malignancy. Many common imaging characteristics are available, such as bone destruction with a moth-eaten to a permeative pattern, a variable amount of mineralized osteoid, various types of periosteal reaction, and adjacent soft tissue mass [23, 24]. Among imaging modalities, MRI is routinely used. Marrow replacement and cortical destruction could be more clearly seen on MRI with heterogeneous signal intensity. It provides excellent tissue contrast, which is much better than computed tomography (CT) and conventional radiography; however, it could not clearly discriminate between OS and EWS due to overlapping imaging characteristics and tumor locations. For instance, most of small cell OS (a subtype of OS) cases own the radiographic features including lytic bone destruction, a soft tissue mass, and periosteal reaction, which are also commonly seen in EWS cases. Furthermore, both their cells are small and have round, hyperchromatic nuclei; they might be mistaken from each other even by histologic analysis [25]. Some advanced sequences to make differential diagnosis have been investigated, such as diffusion-weighted imaging (DWI) [4] and intravoxel incoherent motion (IVIM) [26]. However, the ability of discrimination is not satisfactory.

Radiomics is a new methodology that uses advanced imaging features for differentiation, staging, monitoring tumors, and even detecting tumor genetics. Compared with traditional radiological diagnosis methods, radiomics could provide ample information and improve diagnostic reproducibility. Thus, the combination of radiomics with traditional MRI protocols is worth expecting because it may develop into a model to weaken user dependence of interpretation because the diagnostic accuracy highly depends on the radiologist's experience. Therefore, we decided to evaluate the performance of differentiation ability using radiomic analysis. The value of radiomics has been under investigation for the past decade. Previous studies mainly focused on the establishment of a radiomics nomogram for staging malignant tumors [7], prediction of lymph node metastasis [8, 9], tumor prognosis [10], prediction of treatment response [11], or even prediction of the cancer phenotype [12, 13].

To the best of our knowledge, this study is the first that attempts to use radiomics to differentiate these two osseous malignant tumors. The application we used is a mature platform, and the radiomic features it provides could describe the signal intensity, morphological characteristics, and texture information of the lesions, thereby possibly representing the information of the lesions comprehensively. As demonstrated, radiomics can indicate additional information about the tumor's underlying biology than imaging morphology. For example, in Table 4, the strong associations of High Grey Level Run Emphasis extracted from the T2-FS data set could be explained from the fact that patients possess large and irregular high signal intensity on T2-weighted images in the inner portion of tumors, most likely representing necrotic areas. The presence of these inner, long T2 signal irregular regions suggests that the tumor is rapidly increasing in size and might be more likely to be an OS.

For most oncological MR exams in our institute, T2-FS and CET1 were contained. T2-FS could effectively depict the border of lesions and demonstrate the overall cellular density of lesions to a certain extent, whereas CET1 could describe the vascularity of lesions, reflect the degree of malignancy, and distinguish necrosis and solid tumors. Furthermore, previous studies that used such sequences for radiomic analysis yielded favorable results [27, 28]. Thus, we set up our model using these two sequences. Based on our results, features yielded from T2-FS perform better than those from CET1, with higher AUC and sensitivity, thereby possibly benefiting the patients who are allergic to contrast media. We assumed that the first-order features selected into the T2-FS model evaluate the intralesional heterogeneity, thereby accounting for pathological characteristics that are important for the distinction of OS and EWS. The selection of mainly higher-order features demonstrates that texture heterogeneity in different spatial directions was descriptive of lesion classification.

Overall, the methodology proposed in this work was found to possess high potential for discrimination of OS and EWS. However, it also has some limitations. Firstly, its retrospective nature comparatively reduces the level of evidence. We only analyzed T2-FS and CET1 due to this nature because quite a number of incomplete scans did not contain other advanced sequences, such as DWI. The relatively small data set was another limitation, and a larger patient cohort is needed to create a more robust model. We excluded much data because a part of patients with OS or EWS of the pelvis did not undergo the contrast-enhanced MRI for some reasons, such as claustrophobia, poor compliance, and potential contrast media allergy. Furthermore, some patients' clinical features are incomplete, so we had to exclude this part. Therefore, multivariables of rad-score nomogram establishment were waived regretfully. Moreover, to minimize the image variation, many items of data performed on different 3 T systems were abandoned. Many published radiomic studies divided the patients into the training set and the validation set to validate the performance of the established model. However, because of the small data set, this method needed to be waived. Nonetheless, using the approach for collecting such a scale of data in a single institution has not been easy because of the relatively low morbidity of both diseases, especially only for pelvic lesions. Further research would expect improved performance by using other modalities' images, such as CT, plain films, or some other multiparametric MR images by conducting a prospective, multicenter, rigorous designed study to solve the aforementioned issues. In addition, to a certain extent, using a 2D manual segmentation of tumor (ROI was only drawn on the largest cross-sectional area of the entire lesion) instead of a 3D automatic/semiautomatic method might affect the final result of radiomic feature analysis. Some features, such as lesion volume and irregularity, could not be extracted to be further analyzed. However, because of the strong stability of our selected radiomic features, the difference could be ignored. Moreover, manual segmentation of the tumors by experienced researchers has been applied in many previous studies and yielded excellent results [29–31]. An automatically computerized tumor segmentation method with high reliability should be used in future studies.

## 5. Conclusions

To the best of our knowledge, there is no previously published research in the literature using radiomic analysis to differentiate OS from EWS. In our study, we identified that there was a promising power for the distinction of OS and EWS by using our multiparametric MR-based radiomic analysis.

## Figures and Tables

**Table 1:** Characteristics of patients and tumors.

	Patient characteristics			
	OS	EWS	*t*/*χ*^2^ value	*p* value
No. of patients	35	31		
Age (years)				
Mean ± SD (range)	30.7 ± 16.5 (13–87)	24.5 ± 9.6 (10–66)	1.846^a^	0.069
Gender (M/F)				
Male	19 (47.5%)	21 (52.5%)	1.247^b^	0.264
Female	16 (61.5%)	10 (38.5%)		
Location of tumors				
Ilium	21 (56.8%)	16 (43.2%)	2.472^b^	0.781
Acetabulum	2 (66.7%)	1 (33.3%)		
Pubis	5 (62.5%)	3 (37.5%)		
Ischium	1 (25.0%)	3 (75.0%)		
Sacrum & coccyx	5 (45.5%)	6 (55.5%)		
Soft tissues	1 (33.3%)	2 (66.7%)		

Note: a = *t*-test; b = chi-square test.

**Table 2:** MRI sequence parameters.

Sequence	Plane	Thickness (mm)	Slices	Matrix	TR (msec)	TE (msec)
T2-FS	Axial	7	24	512 × 512	3800–4300	73-85
CET1	Axial	4	108–132	512 × 512	4.3	1.9

**Table 3:** Radiomic features used in the analysis.

Radiomic feature type	References	Radiomic features
Histogram	Sutton [18]	Mean, variance, uniformity, skewness, kurtosis, energy, entropy
Form factor	/	Volume CC, surface, surface volume ratio, compactness, maximum 3D diameter
Haralick and GLCM	Haralick et al. [14]	Entropy, inertia, inverse difference moment
GLRLM	Galloway [15]	Short run emphasis, long run emphasis, gray-level nonuniformity, run-length nonuniformity, run percentage
	Chu et al. [16]	Low gray-level run emphasis, high gray-level run emphasis
	Dasarathy and Holder [17]	Short run low gray-level emphasis, short run high gray-level emphasis, long run low gray-level emphasis, long run high gray-level emphasis

Note: GLCM = gray-level cooccurrence matrix; GLRLM = gray-level run length matrix.

**Table:** (a) Numbers of radiomic feature selection by different methods

	T2-FS	CET1
Independent-sample *t*-test	141	60
Spearman test	27	10
LASSO	9	7

**Table:** (b) Radiomic feature selection by the LASSO method

No.	T2-FS	CET1
1	Intercept	Intercept
2	Correlation_All Direction_offset7_SD	Min intensity
3	Surface volume ratio	Inverse Difference Moment_All Direction_offset 7_SD
4	GLCM Energy_All Direction_offset4_SD	GLCM Entropy_All Direction_offset 1_SD
5	Inverse Difference Moment_All Direction_offset 4_SD	GLCM Energy_angle135_offset 7
6	Inverse Difference Moment_All Direction_offset 7_SD	Volume MM
7	Cluster Prominence_All Direction_offset 7_SD	Surface volume ratio
8	High Grey Level Run Emphasis_All Direction_offset 7_SD	
9	Short Run High Grey Level Emphasis_All Direction_offset 7_SD	

Note: GLCM = gray-level cooccurrence matrix.

**Table 5:** Differentiated performance based on T2-FS and CET1.

	T2-FS	CET1
Sensitivity	74.2%	22.6%
Specificity	82.9%	100%
AUC	0.881 (95% CI: 0.799–0.963)	0.765 (95% CI: 0.652–0.878)

## Data Availability

The datasets used and/or analyzed during the current study are available from the corresponding author on reasonable request.
